# Comparative transcriptome and functional analyses provide insights into the key factors regulating shoot regeneration in highbush blueberry

**DOI:** 10.1093/hr/uhae114

**Published:** 2024-04-22

**Authors:** Masafumi Omori, Hisayo Yamane, Ryutaro Tao

**Affiliations:** Laboratory of Pomology, Graduate School of Agriculture, Kyoto University, Sakyo-ku, Kyoto 606-8502, Japan

## Abstract

Establishing an efficient plant regeneration system is a crucial prerequisite for genetic engineering technology in plants. However, the regeneration rate exhibits considerable variability among genotypes, and the key factors underlying shoot regeneration capacity remain largely elusive. Blueberry leaf explants cultured on a medium rich in cytokinins exhibit direct shoot organogenesis without prominent callus formation, which holds promise for expediting genetic transformation while minimizing somatic mutations during culture. The objective of this study is to unravel the molecular and genetic determinants that govern cultivar-specific shoot regeneration potential in highbush blueberry (*Vaccinium corymbosum* L.). We conducted comparative transcriptome analysis using two highbush blueberry genotypes: ‘Blue Muffin’ (‘BM’) displaying a high regeneration rate (>80%) and ‘O’Neal’ (‘ON’) exhibiting a low regeneration rate (<10%). The findings revealed differential expression of numerous auxin-related genes; notably, ‘BM’ exhibited higher expression of auxin signaling genes compared to ‘ON’. Among blueberry orthologs of transcription factors involved in meristem formation in *Arabidopsis*, expression of *VcENHANCER OF SHOOT REGENERATION* (*VcESR*), *VcWUSCHEL* (*VcWUS*), and *VcCUP-SHAPED COTYLEDON 2.1* were significantly higher in ‘BM’ relative to ‘ON’. Exogenous application of auxin promoted regeneration, as well as *VcESR* and *VcWUS* expression, whereas inhibition of auxin biosynthesis yielded the opposite effects. Overexpression of *VcESR* in ‘BM’ promoted shoot regeneration under phytohormone-free conditions by activating the expression of cytokinin- and auxin-related genes. These findings provide new insights into the molecular mechanisms underlying blueberry regeneration and have practical implications for enhancing plant regeneration and transformation techniques.

## Introduction

Plant cells possess a remarkable inherent potential to give rise to a complete plant from somatic cells, a phenomenon known as totipotency [1–3]. Establishing an efficient plant regeneration system is a crucial prerequisite for the application of genetic engineering techniques in plants. While genetic transformation and genome editing technologies have been utilized for breeding horticultural crops, a lower *in vitro* regeneration rate remains a significant obstacle for genetic engineering in numerous plant species [4]. Hence, the development of an efficient and reliable regeneration system is imperative.

Various pathways exist for *in vitro* plant regeneration. Indirect regeneration (callus-mediated regeneration) and somatic embryogenesis are the most commonly employed approaches. However, both methods are time-consuming and require numerous subcultures to achieve regenerants. For example, callus-mediated regeneration typically involves a two-step process, commencing with totipotency induction in an auxin-rich medium and subsequent shoot regeneration in a cytokinin-rich medium [5, 6]. In contrast, direct regeneration is a one-step process that bypasses the callus stage [7]. Direct regeneration holds promise for micropropagation or transformation, as it typically requires less time to initiate regeneration compared to the callus-mediated regeneration system. Moreover, direct regeneration has the potential to minimize detrimental somatic mutations that often arise during callus culture [8], leading to regeneration with minimal alterations in the plant genome. Notably, in certain plant species, plants derived through direct regeneration exhibit greater genetic fidelity compared to those obtained through indirect regeneration [9–11]. Although direct regeneration has been documented in several plant species [12–14], the underlying molecular mechanisms governing these processes remain largely unknown.

A previous study reported a successful protocol for callus-mediated regeneration and transformation of blueberries [15, 16]. However, there is still a demand for a rapid regeneration and transformation system. Direct regeneration has been commonly observed in blueberries when explants were cultured in a cytokinin-containing medium devoid of auxin [17, 18]. Georgieva [19] investigated the regeneration capacity of six highbush blueberry cultivars, and the rate of direct regeneration ranged from 10 to 80%, suggesting a significant influence of genotype on shoot regeneration capacity. Regeneration in an auxin-free medium represents a desirable trait for plant transformation studies and breeding, as the exogenous application of auxin in plant tissue culture may induce unintended somaclonal variations [20, 21]. Despite extensive previous research on the effects of genotypes or culture conditions on shoot regeneration, no studies have explored the genetic mechanisms underlying shoot regeneration in blueberry.

The objective of this study is to elucidate the crucial molecular mechanisms and genetic factors that govern shoot regeneration capacity in highbush blueberry. We employed two highbush blueberry cultivars, ‘Blue Muffin’ (‘BM’) and ‘O’Neal’ (‘ON’), which exhibited divergent regeneration capacities. The comparative histological and molecular characterization, along with functional gene evaluation conducted in this study, provides novel insights into the molecular basis of blueberry regeneration, thus contributing to the advancement of plant biotechnology methods.

## Results

### Comparative characterization of plant regeneration ability in highbush blueberry cultivars

**Figure 1:**
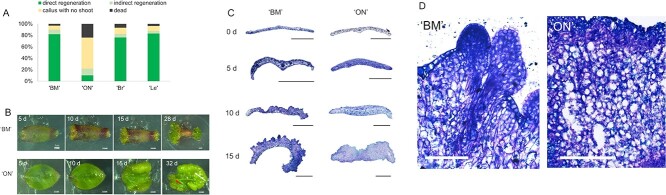
Characterization of direct shoot regeneration from highbush blueberry leaf explants. (A) Regeneration rate of four highbush blueberry cultivars. Leaf explants were placed on the 1.0 mg/l TDZ medium. Three independent experiments with 20 leaf explants for each medium were performed. Direct regeneration refers to shoot regeneration without obvious callus formation, while indirect regeneration indicates shoot regeneration via callus formation. ‘Br’: ‘Brigitta’, ‘Le’: ‘Legacy’. (B) Shoot regeneration in ‘BM’ leaf explants and callus formation in ‘ON’ explants. Leaf explants were placed on the 1.0 mg/l TDZ medium. ‘BM’ explants exhibited direct regeneration, while most ‘ON’ explants formed callus (indicated by the white arrow), and some explants formed meristem (indicated by the red arrow). (C) Histological observations of transverse sections of ‘BM’ and ‘ON’ leaf explants. Leaf explants were placed on the 1.0 mg/l TDZ medium. Scale bars = 500 μm. (D) Cytological observation of transverse sections of ‘BM’ and ‘ON’ 10-day leaf explants. In ‘BM’, the epidermis was elevated due to increased cell number and longitudinal cell elongation. In ‘ON’, the cells were relatively rounded and exhibited proliferative growth without a specific orientation. Scale bars = 100 μm.

Leaf explants from four highbush blueberry cultivars, ‘BM’, ‘ON’, ‘Brigitta’, and ‘Legacy’, were cultured with the abaxial side facing downward on the regeneration medium (MW basal medium [22] supplemented with 20 g/l sucrose, 1.0 mg/l thidiazuron (TDZ), and 6 g/l agar). The explants were categorized into four groups: direct regeneration, indirect regeneration, callus with no shoots, and dead, at 40 days after culture. Approximately 80% of ‘BM’, ‘Brigitta’, and ‘Legacy’ explants directly regenerated vigorous adventitious shoots at 1 month after initiating culture incubation on the TDZ medium (Fig. 1A and B). In contrast, most ‘ON’ explants on the TDZ medium exhibited callus formation without shoots (Fig. 1). Around 20% of ‘ON’ explants showed adventitious bud formation through direct or indirect regeneration but did not exhibit visible shoot formation. The regeneration rate of ‘ON’ was less than 20% regardless of the type and concentration of cytokinin, indicating that the regeneration potential of ‘ON’ was significantly lower compared to the other three cultivars. Therefore, ‘BM’ with a high regeneration capacity and ‘ON’ with a low regeneration capacity were selected for further observations and analysis.

Morphological differences in explants between ‘BM’ and ‘ON’ were observed. The initial anatomical changes in the two cultivars involved cell division, particularly in subepidermal cells (Fig. 1C). Most explants exhibited anticlinal cell division at ~5 days after excision and placement on the medium. At 5 days, the epidermis of ‘BM’ appeared slightly bumpy, while ‘ON’ had a smooth epidermis. By 10 days, active cell division was observed in the upper part of the vascular bundles in ‘BM’. Meristem structures were also observed in the majority of ‘BM’ explants by 10 days. In contrast, in ‘ON’, cell division was active but with a flat shape, and only a few apical meristem-like structures were observed. At 15 days, ‘BM’ exhibited the formation of adventitious buds, including leaf primordia and meristems. ‘ON’ displayed a callus-like structure with few meristems. In ‘BM’, the epidermis was elevated due to increased cell numbers and longitudinal cell elongation (Fig. 1D). In ‘ON’, the cells were relatively round and seemed to proliferate without a definite direction (Fig. 1D).

### Global changes in gene expression in leaf explants during regeneration

To elucidate the molecular and genetic mechanisms that confers cultivar-specific shoot regeneration potential in highbush blueberry, we conducted comparative transcriptome analysis using two representative highbush blueberry genotypes, ‘BM’ and ‘ON’, which exhibited contrasting regeneration capacities. RNA was extracted from the leaves of *in vitro* shoots (0 days) and explants on 1.0 mg/l TDZ medium collected at 2, 4, and 8 days after culture. For RNA-seq in this study, a reference genome consisting of 55 648 representative genes selected from 128 559 genes in the tetraploid ‘Draper’ genome was utilized [23, 24]. Principal component analysis (PCA) indicated that a significant change in the gene expression profile occurred at 2 days postculture (2 d) in both cultivars (Supplementary Fig. S1). The global gene expression changes appeared to be similar between the two cultivars from 2 to 8 days, with more pronounced changes observed in ‘BM’. Gene ontology (GO) terms related to ribosomes, protein synthesis, nucleoli, and stress response were enriched in the upregulated differentially expressed genes (DEGs) from 0 to 2 days in both cultivars (Supplementary Tables S1 and S2). This may reflect typical responses immediately after tissue culture and corresponds to a preparatory stage for dedifferentiation and cell division [14].

**Table 1:** GO analysis for upregulated DEGs in ‘BM’ 8 days compared with ‘ON’ 8 days

Category	Term	Ontology	DEG	Total gene	*P* value	FDR
GO:0009734	Auxin-activated signaling pathway	BP	55	362	1.40E−11	1.20E−07
GO:0048825	Cotyledon development		12	44	9.00E−06	9.90E−03
GO:0006725	Cellular aromatic compound metabolic process		6	12	1.30E−05	9.90E−03
GO:0030001	Metal ion transport		14	76	4.60E−05	2.70E−02
GO:0016042	Lipid catabolic process		34	274	5.30E−05	2.70E−02
GO:0010103	Stomatal complex morphogenesis		6	15	1.90E−04	7.70E−02
GO:0010075	Regulation of meristem growth		7	22	2.40E−04	9.30E−02
GO:0006355	Regulation of transcription, DNA-templated		98	1118	2.50E−04	9.30E−02
GO:0000786	Nucleosome	CC	18	85	3.10E−08	1.20E−04
GO:0005576	Extracellular region		120	1360	3.00E−06	5.00E−03
GO:0031225	Anchored component of membrane		36	281	8.10E−06	9.90E−03
GO:0005618	Cell wall		73	775	6.10E−05	2.90E−02
GO:0046982	Protein heterodimerization activity	MF	30	195	4.30E−08	1.20E−04
GO:0003700	DNA-binding transcription factor activity		162	1843	6.70E−08	1.40E−04
GO:0008807	Carboxyvinyl-carboxyphosphonate phosphorylmutase activity		5	7	1.10E−05	9.90E−03
GO:0102387	2-Phenylethanol acetyltransferase activity		6	10	1.30E−05	9.90E−03
GO:0102720	Acetyl-coenzyme A:acetyl alcohol acetyltransferase activity		6	10	1.30E−05	9.90E−03
GO:0003677	DNA binding		146	1739	1.40E−05	9.90E−03
GO:0042973	Glucan endo-1,3-beta-D-glucosidase activity		16	78	2.30E−05	1.50E−02
GO:0016788	Hydrolase activity, acting on ester bonds		22	147	5.10E−05	2.70E−02
GO:0046566	DOPA dioxygenase activity		5	9	6.70E-05	2.90E−02
GO:0050297	Stizolobate synthase activity		5	9	6.70E-05	2.90E−02

GO terms with FDR < 0.05 were listed.

K-means clustering and GO enrichment analysis were performed for each cluster to gain an overview of how shoot regeneration occurred in ‘BM’. Two thousand genes with large expression variation among stages were classified into six clusters based on their expression patterns (Supplementary Fig. S2), and GO analysis was conducted for each cluster. GO terms related to photosynthesis and chloroplasts were significantly enriched in Cluster A (Supplementary Table S3), which exhibited a sharp decrease in expression at 2 days. This suggests that the explants lost their characteristics as leaves with the initiation of culture. GO terms related to cell wall catabolic processes and damage response were enriched in Cluster C (Supplementary Table S4), which showed a rapid increase in expression at 2 days and a subsequent rapid decrease at 4 days. Cluster D, which maintained high expression from 2 to 8 days, exhibited GO terms related to flavonoid synthesis, primary cell wall, and cytokinin signaling (Supplementary Table S5). GO terms related to cell division, cell wall organization, and organogenesis were accumulated in Cluster F (Supplementary Table S6), which increased from 2 to 8 days and peaked at 8 days. Collectively, these results allowed us to propose the regeneration process in ‘BM’. Specifically, after culture initiation, the cells were first stimulated by wound and cytokinin responses (0–2 days after culture), followed by cell wall reorganization and activation of cell division (4–8 days after culture), ultimately leading to meristem formation (10 days after culture).

### Auxin signaling and transport may play an important role in ‘BM’-specific high regeneration capacity

GO enrichment analysis using significantly upregulated genes in ‘BM’ compared to ‘ON’ at each stage revealed factors involved in the genotype-specific regeneration ability in blueberry. No significant GO term was detected at 0 day, and only carboxyvinyl-carboxyphosphonate phosphorylmutase activity was enriched at 2 days. No previous research was found to investigate the relation between this GO term and regeneration. At 4 days, enriched GO terms included stomatal development, cell division, and microtubules. At 8 days, the auxin-activated signaling pathway was the most highly enriched, along with GO terms such as nucleosome, transcription factors, and meristem growth (Table 1). Genes related to these GO terms were further analyzed.

**Figure 2:**
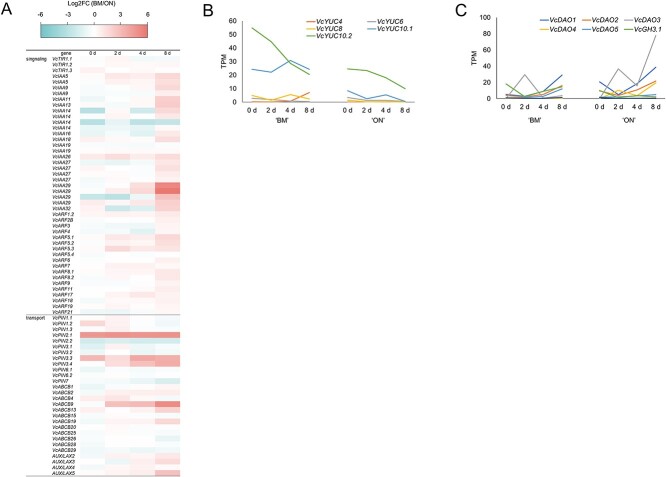
Expression levels of auxin-related genes in ‘BM’ and ‘ON’ leaf explants. (A) Heatmap illustrating log2FC(‘BM’/’ON’) of auxin signaling and transport genes. Genes include *VcTRANSPORT INHIBITOR RESPONSE 1* (*VcTIR1*), *VcAUXIN RESPONSE FACTOR* (*VcARF*), *VcPIN-FORMED* (*VcPIN*), *VcATP-BINDING CASSETTE subfamily B* (*VcABCB*), and *VcAUXIN RESISTANT 1/LIKE AUX1* (*VcAUX/LAX*). Genes with TPM > 1 were selected in any of the cultivars or stages. (B) Expression changes of auxin biosynthesis gene, *VcYUCCA* (*VcYUC*), in ‘BM’ and ‘ON’ after starting culture. Genes with TPM > 1 were selected in any of the cultivars or stages. (C) Expression changes of auxin metabolism genes, *VcDIOXYGENASE OF AUXIN OXIDATION* (*VcDAO*), and *VcGRETCHEN HAGEN 3* (*VcGH3*), in ‘BM’ and ‘ON’ after starting culture. Genes with TPM > 1 were selected in any of the cultivars or stages.

Based on the GO enrichment analysis, we further analyzed expression of genes related to auxin individually. The GO term ‘auxin-signaling pathway’ encompasses genes involved in auxin signaling and transport. The expression of most auxin signaling genes *VcTRANSPORT INHIBITOR RESPONSE 1* (*VcTIR1*), *VcAUXIN RESPONSE FACTOR*s (*VcARF*s), and *VcINDOL-3-ACETIC ACID INDUCIBLE* (*VcIAA*) was higher in ‘BM’ compared to ‘ON’ at 8 days (Fig. 2A). Among the auxin transport genes [*VcPIN-FORMED* (*VcPIN*), *VcATP-BINDING CASSETTE subfamily B* (*VcABCB*), and *AUXIN RESISTANT 1/LIKE AUX1* (*VcAUX/LAX*)], six genes (*VcPIN2.1*, *VcPIN3.3*, *VcPIN3.4*, *VcABCB9*, *VcABCB13*, *VcAUX/LAX5*) exhibited significantly higher expression in ‘BM’ compared to ‘ON’ at 8 days, while one gene (*VcPIN2.2*) showed lower expression (Fig. 2A). The expression level of *YUCCA* genes (*VcYUC*), which encode the rate-limiting enzyme for indole-3-acetic acid (IAA) biosynthesis [25], was higher in ‘BM’ (Fig. 2B). Other biosynthetic enzyme genes, *VcTRYPTOPHAN AMINOTRANSFERASE RELATED* (*VcTAR*), showed similar expression levels and patterns in both cultivars, while *VcTRYPTOPHAN AMINOTRANSFERASE OF ARABIDOPSIS1* (*VcTAA*) genes exhibited very low expression levels (Transcript per million (TPM) < 1) in both cultivars. The expression of auxin metabolism genes [*VcDIOXYGENASE OF AUXIN OXIDATION* (*VcDAO*) and *VcGRETCHEN HAGEN 3* (*VcGH3*)], which inactivate IAA, was higher in the ‘ON’ cultivar (Fig. 2C). In summary, substantial differences were observed in the expression of auxin-related genes between the cultivars.

**Figure 3:**
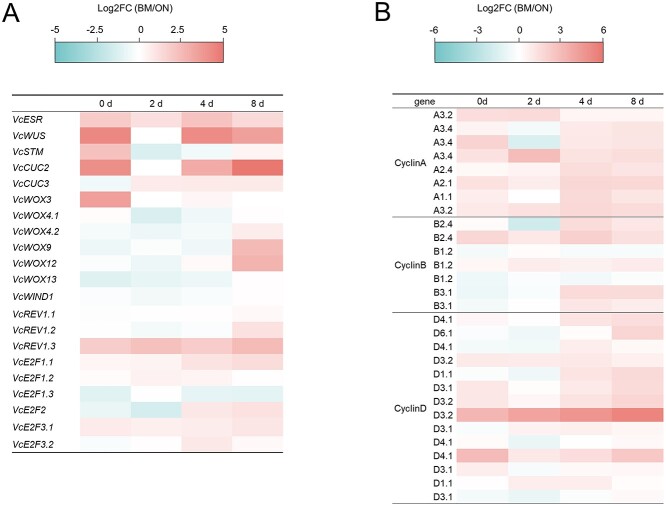
Expression levels of transcription factor and cyclin genes in ‘BM’ and ‘ON’ leaf explants. (A) Expression changes of transcription factor genes, *VcENHANCER OF SHOOT REGENERATION* (*VcESR*), *VcWUSCHEL* (*VcWUS*), *VcSHOOT MERISTEMLESS* (*VcSTM*), and *VcCUP-SHAPED COTYLEDON 2*, *3* (*VcCUC2*, *3*) in ‘BM’ and ‘ON’ after starting culture. Genes with TPM > 1 were selected in any of the cultivars or stages. (B) Heatmap illustrating log2FC(‘BM’/’ON) of cyclin genes. Genes with TPM > 1 were selected in any of the cultivars or stages.

We also closely examined the expression levels of transcription factors known to be associated with stem cell maintenance and meristem formation in Arabidopsis [26]. Among them, the expression of *VcESR*, *VcWUS*, and *VcCUC2*, was especially higher in ‘BM’ compared to ‘ON’ (Fig. 3A). The expression of cyclin genes (*VcCYCA*, *VcCYCB*, and *VcCYCD*), which can be activated by phytohormones and environmental signals and regulated the cell cycle [27, 28], was higher in ‘BM’ than in ‘ON’ (Fig. 3B). These findings suggested that cell division is more active in ‘BM’ than in ‘ON’.

**Figure 4:**
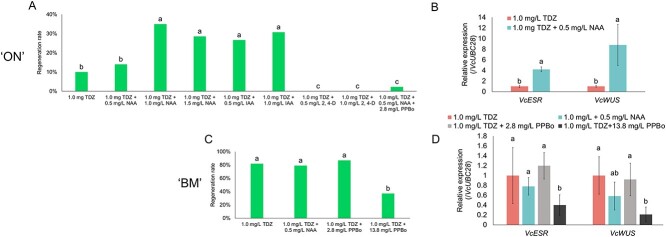
Effects of exogenous auxin and auxin inhibitor on the regeneration rate and regeneration-related gene expression in ‘ON’ and ‘BM’. (A) The effect of exogenous auxin and auxin inhibitor on the regeneration rate of ‘ON’ explants. Two independent experiments with 20 leaf explants for each medium were performed. Chi-square test for multiple comparisons was used to detect significant differences among media (*P* < 0.05). (B) The effect of exogenous auxin on regeneration-related gene expression in ‘ON’ explants. 10–20 leaves were pooled as one biological replicate for RNA extraction. qPCR analysis was performed with three biological replicates and two technical replicates. Student’s *t*-test was used to detect significant differences between TDZ and TDZ + NAA (*P* < 0.05). (C) The effect of exogenous auxin inhibitor on the regeneration rate of ‘BM’ explants. Two independent experiments with 20 leaf explants for each medium were performed. Chi-square test for multiple comparisons was used to detect significant differences among media (*P* < 0.05). (D) The effect of exogenous auxin inhibitor on regeneration-related gene expression in ‘BM’ explants. Three biological replicates and two technical replicates were analyzed. Significant differences in gene expressions among the different media were determined by Tukey’s HSD test (*P* < 0.05).

Our transcriptome analysis indicated that the auxin-activated signaling pathway could be involved in the high regeneration capacity of ‘BM’, which prompted us to explore the effects of auxin on regeneration in blueberry. The regeneration rate of ‘ON’ was significantly higher when 1-Naphthalene acetic acid (NAA) and IAA were supplemented on TDZ media compared to TDZ media. No regeneration was observed in the media containing 2,4-Dichlorophenoxyacetic acid (2,4-D). Additionally, the expression levels of *VcESR* and *VcWUS* were significantly higher in 1.0 mg/l TDZ + 0.5 mg/l NAA media compared to 1.0 mg/l TDZ media (Fig. 4A, B). The supplementation of 2.8 mg/l 4-phenoxyphenylboronic acid (PPBo) to 1.0 mg/l TDZ + 0.5 mg/l NAA medium significantly reduced the regeneration rate in ‘ON’ (Fig. 4A). PPBo targets the IAA biosynthesis enzyme (YUCCA) and inhibits its activity [29]. In contrast to ‘ON’, in ‘BM’, TDZ medium produced high *VcESR* and *VcWUS* expression levels comparable to TDZ + NAA medium, which suggested that exogenous auxin is not required to increase those gene expressions. No significant differences were observed in the regeneration rate and the expression of *VcESR* and *VcWUS* between TDZ media and TDZ + 2.8 mg/l PPBo while the addition of 13.8 mg/l PPBo to TDZ media significantly decreased the regeneration rate and the expression levels of *VcESR* and *VcWUS*. These results suggested that auxin activated *VcESR* and *VcWUS* expression, thereby promoting regeneration.

Since cytokinin is also known to promote regeneration, we investigated gene expression changes of cytokinin biosynthesis, activation, receptor, signaling, and metabolic pathway genes. As shown in Supplementary Fig. S3, no consistent differences were found between ‘BM’ and ‘ON’.

### Overexpression of *VcESR* in blueberry

**Figure 5:**
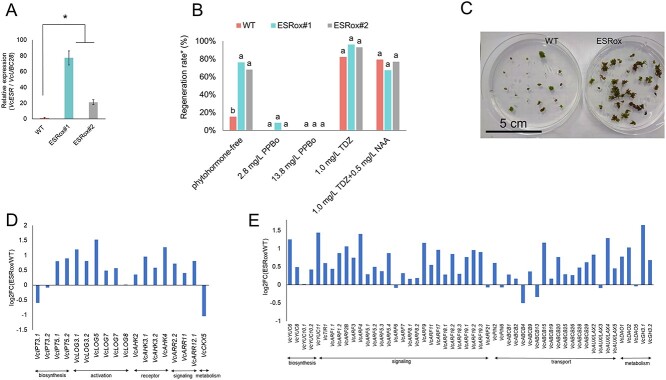
Analysis of *VcESR* overexpressing ‘BM’ transgenic plants. (A) *VcESR* expression in the leaf of wild type (WT) and transgenic lines (ESRox#1, 2). Three biological replicates and two technical replicates were analyzed. Significant differences in gene expressions among the different media were determined by Tukey’s honest significant difference (HSD) test (*P* < 0.05). (B) Shoot regeneration rate of WT and transgenic lines. Two independent experiments with 20 leaf explants for each line and medium were performed. Chi-square test for multiple comparisons was used to detect significant differences among the lines for each medium (*P* < 0.05). (C) Phytohormone-independent shoot regeneration in explants of ESRox#1. (D) Comparison of cytokinin-related gene expression between WT and ESRox#1. Genes with TPM > 1 were selected. (E) Comparison of auxin-related gene expression between WT and ESRox#1. Genes with TPM > 1 were selected.

Finally, we aimed to clarify the role of *VcESR* by using *VcESR*-overexpressing blueberry. VcESR has conserved AP2/ERF domain for DNA binding and ESR motif [30] for transactivation (Supplementary Fig. S4A). According to phylogenetic analysis using ESR family genes, VcESR is closer to ESR2 than ESR1 (Supplementary Fig. S4B). Arabidopsis *ESR1* and *ESR2* both enhanced shoot regeneration and their expressions are differentially regulated [31]. Two ‘BM’ lines transformed with pPLV26-35S:VcESR were obtained and named ESRox#1 and #2. The expressions of *VcESR* in the leaves of *in vitro* shoots were significantly higher in the transgenic lines compared to the wild type (WT) (Fig. 5A). When leaves of the transgenic lines were placed on a phytohormone-free medium, much higher regeneration rates were observed compared to WT (Fig. 5B and C). In contrast, only a few WT explants exhibited regeneration without phytohormone. Therefore, we confirmed that *VcESR* promoted shoot regeneration independent of exogenous phytohormone treatment. Supplementation of PPBo to phytohormone-free medium dramatically decreased the regeneration rate of ESRox#1 and 2, suggesting that regeneration without exogenous phytohormone by *VcESR* overexpression is dependent on auxin biosynthesis.

To explore how ESRox#1 could regenerate under phytohormone-free conditions, RNA-seq analysis was performed. RNA was extracted from leaves of WT and ESRox#1 in vitro shoots. The log2FC (ESRox#1/WT) values of cytokinin and auxin-related genes are shown in Fig. 5D and E. Most of the cytokinin and auxin-related genes exhibited higher expression levels than WT, indicating that *VcESR* overexpression enhanced regeneration ability by activating cytokinin and auxin-related genes.

## Discussion

Auxin is necessary for acquiring pluripotency for shoot regeneration during the initial stages of culture [32]. In the two-step regeneration system of *Arabidopsis*, explants are first incubated on an auxin-rich medium and then transferred to a cytokinin-rich medium to acquire regeneration competence. However, the response to auxin and its role in plant regeneration vary among plant species and genotypes. In blueberry, some cultivars exhibit auxin-free one-step regeneration in a genotype-dependent manner [33]. Here, our aim was to identify the genetic factors that control direct shoot regeneration in blueberry, with a particular focus on cytokinin and auxin. Our study collectively suggested that auxin signaling and transcription factors related to meristem formation may play important roles in shoot regeneration and contribute to genotype-specific shoot regeneration capacity in blueberry.

Our transcriptome analysis revealed that cytokinin-related genes were highly expressed during shoot regeneration in ‘BM’. Indeed, cytokinin application was necessary for the regeneration process in blueberry [16]. However, no consistent differences were found in cytokinin-related gene expression between ‘BM’ and ‘ON’. This suggested that cytokinin metabolism and signaling may not be crucial factors underlying the high regeneration capacity specific to ‘BM’. On the other hand, our transcriptome analysis also suggested that auxin signaling may have important roles in the high regeneration rate specific to ‘BM’. Auxin regulates various aspects of plant growth and development, including cell division, expansion, elongation, and differentiation [34]. ‘BM’ exhibited increased cell numbers and longitudinal cell elongation prior to shoot regeneration, while ‘ON’ did not. In liverwort (*Marchantia polymorpha*), mutants of auxin signaling genes (*tir1*, *arf1*) exhibited defects in cell division patterns and meristem formation, indicating that auxin signaling regulated formative cell divisions, leading to stem cell formation and apical growth [35, 36]. We also observed differences in sensitivity to auxin inhibitor treatment between ‘BM’ and ‘ON’. The 2.8 mg/l PPBo treatment significantly reduced the regeneration rate in ‘ON’, whereas in ‘BM’, the 2.8 mg/l PPBo treatment did not affect the regeneration rate, but the 13.8 mg/l PPBo treatment significantly reduced it. Therefore, we hypothesize that differences in auxin signaling and/or auxin metabolism may underlie the genotype-specific shoot regeneration capacity in blueberry.

Cytokinin and auxin regulate the expression of various transcription factors to promote regeneration [37]. For instance, *ESR1/DORNRÖSCHEN* could induce cytokinin-independent shoot formation from root cultures in Arabidopsis [38]. Upregulation of *ESR1* driven by a chemical-inducible promoter stimulated shoot regeneration in *Arabidopsis* [38, 39]. Furthermore, overexpression of an *ESR1* ortholog (*EARLY BUD-BREAK 1*; *EBB1*) in hybrid poplar (*Populus tremula* × *P. alba*) doubled the shoot regeneration rate during transformation [40]. In this study, we demonstrated that *VcESR* overexpression also promoted regeneration in blueberry, suggesting that the regeneration-promoting effect of the *ESR* family may be conserved across species. Comparative transcriptome analysis between the WT and the *VcESR*-overexpressing transgenic line revealed higher expression of cytokinin and auxin-related genes in the transgenic lines than in WT. Under phytohormone-free conditions, the transgenic plants were able to regenerate by regulating phytohormone-related genes. Transcriptome analysis of poplar (*Populus trichocarpa*) dormant buds overexpressing peach *EBB1* (*PpEBB1*) showed that *PpEBB1* promoted bud break by regulating cell division, cell wall modification, and phytohormones such as auxin and cytokinin [41]. Enhancement of regeneration by ESR homologs has been also reported in rice [42] and in liverwort [43]. The genetic mechanisms regulated by *ESR* orthologs that promote organogenesis may also be conserved in the plant kingdom. The utilization of *VcESR* holds the promise to develop a genotype-independent transformation system [44], which is useful for molecular breeding for crop improvement in transformation-recalcitrant species and cultivars. We tried to generate transgenic ‘ON’ plants overexpressing *VcESR* to validate that the absence of *VcESR* upregulation is the cause of low regeneration ability. However, we could not get any ‘ON’ transformants probably because of low susceptibility to Agrobacterium. Genotype-independent transformation will be achieved by combination of regeneration promoter such as *VcESR* and improvement of susceptibility to Agrobacterium.

Finally, we propose that changes in *VcESR* expression during adventitious bud formation may be influenced by cytokinin and auxin in the medium. In ‘ON’, *VcESR* was significantly highly expressed in TDZ + NAA medium than in TDZ medium, indicating that cytokinin and auxin synergistically activated *VcESR* expression. This result was consistent with previous studies. Banno *et al*. [38] demonstrated that cytokinins induced *ESR1* expression only after preincubation with 2,4-D, a type of auxin, in *Arabidopsis* root explants. Iwase *et al*. [45] indicated that *ESR1* promoter activity was more strongly stimulated by concurrent application of cytokinin and auxin than by cytokinin or auxin alone. In hybrid poplar, *EBB1* was induced by a combination of cytokinin and auxin treatments, whereas treatment with cytokinin or auxin alone did not promote *EBB1* expression [39]. Interestingly, ‘BM’ exhibited high *VcESR* expression induced by TDZ, which was comparable to expression induced by TDZ + NAA. Additionally, *VcESR* expression in ‘BM’ on TDZ medium was higher than that in ‘ON’. The results of auxin treatment indicated that ‘BM’ may have the potential to exhibit high *VcESR* expression regardless of exogenous auxin treatment. Moreover, the results suggested that high amount of endogenous auxin content in ‘BM’ could compensate exogenous auxin application. The expression levels of *VcESR* could be used as biomarkers for endogenous auxin levels, auxin sensitivity, and regeneration ability. However, further studies are necessary to validate this possibility.

## Materials and methods

### Characterization of regeneration capacity in highbush blueberries

Four highbush blueberry cultivars, namely ‘BM’, ‘ON’, ‘Brigitta’, and ‘Legacy’, were employed as plant materials. The in vitro shoots were propagated in a shoot proliferation medium composed of MW basal medium supplemented with 20 g/l sucrose, 1.1 mg/l zeatin, and 6 g/l agar. The MW medium consisted of equal volumes of MS [46] and WPM [47] media. At least 40 leaf explants from each of the four cultivars were cultured with the abaxial side facing downward on the regeneration medium (MW supplemented with 20 g/l sucrose, 1.0 mg/l TDZ, and 6 g/l agar). In the case of ‘ON’, four different conditions with varying cytokinins and concentrations (1.0 mg/l TDZ, 2.0 mg/l TDZ, 1.1 mg/l zeatin, and 4.4 mg/l zeatin) were tested as regeneration media. The explants were categorized into four groups: direct regeneration, indirect regeneration, callus with no shoots, and dead, at 40 days after culture. The pH of all media was adjusted to 5.2 before sterilization in an autoclave at 121°C for 20 minutes. All tissue-cultured plants were maintained at 24°C with a 16-hour photoperiod (30 mE/m2/s from cool white fluorescent tubes).

### Histological observation of regeneration processes

Leaf explants were collected at 0, 5, 10, and 15 days after culture. The samples were fixed in formalin–acetic acid–ethanol and then subjected to a sucrose gradient (10% and 20%) before embedding in SCEM embedding medium (Leica Microsystems GmbH, Wetzlar, Germany). Frozen samples were sectioned into 10 μm slices using a CM1520 cryostat (Leica) following the method by Kawamoto [48]. The cross-sections were stained with 0.5% toluidine blue and examined and photographed under a BX60 light microscope (Olympus Corporation, Tokyo, Japan) equipped with a digital camera (DP72 LPT, Olympus).

### RNA-seq analysis

RNA was extracted from the leaves of *in vitro* shoots (0 days) and explants on TDZ medium collected at 2, 4, and 8 days after culture. To obtain sufficient RNA volume, 10–20 leaves were pooled as one biological replicate for RNA extraction. The collected samples were immediately frozen with liquid nitrogen and stored at −80°C. Total RNA was isolated from the frozen samples using PureLink™ Plant RNA Reagent (Invitrogen, Carlsbad, CA, USA). All transcriptome analyses were performed with three biological replicates.

For mRNA-seq, libraries were constructed and sequenced using BGI-SEQ with 200 bp paired reads, following the manufacturer’s instructions. The sequencing reads were initially trimmed using Fastp [49] with default parameters for the removal of adapter sequences and low-quality bases (quality score <15). In this study, a reference genome consisting of 55 648 representative genes selected from 128 559 genes in the tetraploid ‘Draper’ genome was utilized [23, 24]. The clean reads were mapped to the aforementioned reference transcriptome using Bowtie2 (version 2.3.5.1) [50]. The TPM values for each transcriptome data were calculated using the rsem-calculate-expression program implemented by RSEM software (version 1.3.1) [51]. The TPM values for each sample were subjected to PCA using the R prcomp function. K-means clustering analysis was performed on the iDEP website [52] (http://bioinformatics.sdstate.edu/idep96/). DEGs between stages and cultivars were identified using the DESeq2 package [53] with a threshold of adjusted FDR ≤ 0.05 and fold-change ≥2. Gene Ontology (GO) terms were assigned to each gene based on functional annotation using the UniprotKB database (https://www.uniprot.org/). GO enrichment analysis was performed using the GOseq2 R package [54]. GO terms with an adjusted FDR ≤ 0.05 were considered significantly enriched.

### Auxin and auxin inhibitor treatment and quantitative PCR (qPCR) analysis

Regeneration medium containing 1.0 mg/l TDZ was supplemented with 0.5, 1.0, and 1.5 mg/l NAA, 0.5 and 1.0 mg/l IAA, 0.5 mg/l 2,4-D, and 2.8 and 13.8 mg/l PPBo. The regeneration rate for each medium was recorded at 40 days after culture. A chi-squared test for multiple comparisons was used to detect significant differences among the different media.

Leaf explant samples on the aforementioned media were collected 8 days after culture. RNA extraction was performed as described above. qPCR was performed as described previously [55]. Primer sequences are listed in Supplementary Table S7. They were designed in the conserved region of all alleles of *VcESR* and *VcWUS*. The relative expression levels of *VcESR* and *VcWUS* were calculated according to the 2^(−∆∆Ct) method using the blueberry *UBIQUITIN CONJUGATING ENZYME 28* (*VcUBC28*) gene as an internal reference. Significant differences in gene expressions among the different media were determined by Tukey’s honest significant difference (HSD) test (*P* < 0.05). Three biological replicates and two technical replicates were analyzed.

### Construction of *VcESR* overexpressing vector

The full-length coding sequence of the blueberry ESR ortholog (VcESR, VaccDscaff2-processed-gene-253.2-mRNA-1) was amplified from ‘ON’ leaf DNA using PrimeStar GXL (TaKaRa Bio Inc., Kusatsu, Japan). The primers for cloning *VcESR* are listed in Supplementary Table S1. The amplified fragment was inserted into the HpaI-digested pPLV26 vector using the In-Fusion HD cloning kit (In-Fusion® HD Cloning Kit, TaKaRa Bio Inc.). The *VcESR* sequence was placed under the Cauliflower mosaic virus 35S promoter. The resulting plasmid vector, pPLV26-35S:VcESR, was verified by Sanger sequencing and then transformed into *Agrobacterium tumefaciens* strain EHA105 by electroporation, along with the helper vector pSOUP.

### Genetic transformation of blueberries

Leaf explants of ‘BM’ were transformed as previously described [16]. The genomic integration of transgenes was confirmed by PCR. Primers amplifying partial sequences of the *neomycin phosphotransferase II* (*nptII*) gene were designed and used for the PCR analysis. Detailed information regarding the primers is provided in Supplementary Table S7.

### Characterization of regeneration capacity of the transgenic line

Total RNA was extracted from the leaves of the WT and the transgenic *in vitro* plant. The RNA was subjected to qPCR and RNA-seq analysis. Tukey’s HSD test was used to detect significant differences in *VcESR* expression levels between WT and the transgenic lines. Subsequently, at least 40 leaves of WT and the transgenic lines were placed on three different media (phytohormone-free, 1.0 mg/l TDZ, 1.0 mg/l TDZ + 0.5 mg/l NAA medium). The regeneration rate was assessed at 40 days after culture. Tukey–Krammer test was used to determine significant differences in regeneration rate between WT and the transgenic lines.

## Supplementary Material

Web_Material_uhae114
