# Supplementary material for: Comparative transcriptome and functional analyses provide insights into the key factors regulating shoot regeneration in highbush blueberry
Source: Hortic Res. 2024 Apr 22;11(6):uhae114. doi: 10.1093/hr/uhae114 (PMC11197304; doi:10.1093/hr/uhae114)
Supplement: Web_Material_uhae114 [file web_material_uhae114.docx]

**Supplementary Table 1. Top 20 GO terms detected by GO enrichment analysis of up-regulated genes in 2 d compared with in 0 d in ‘BM’.**

**
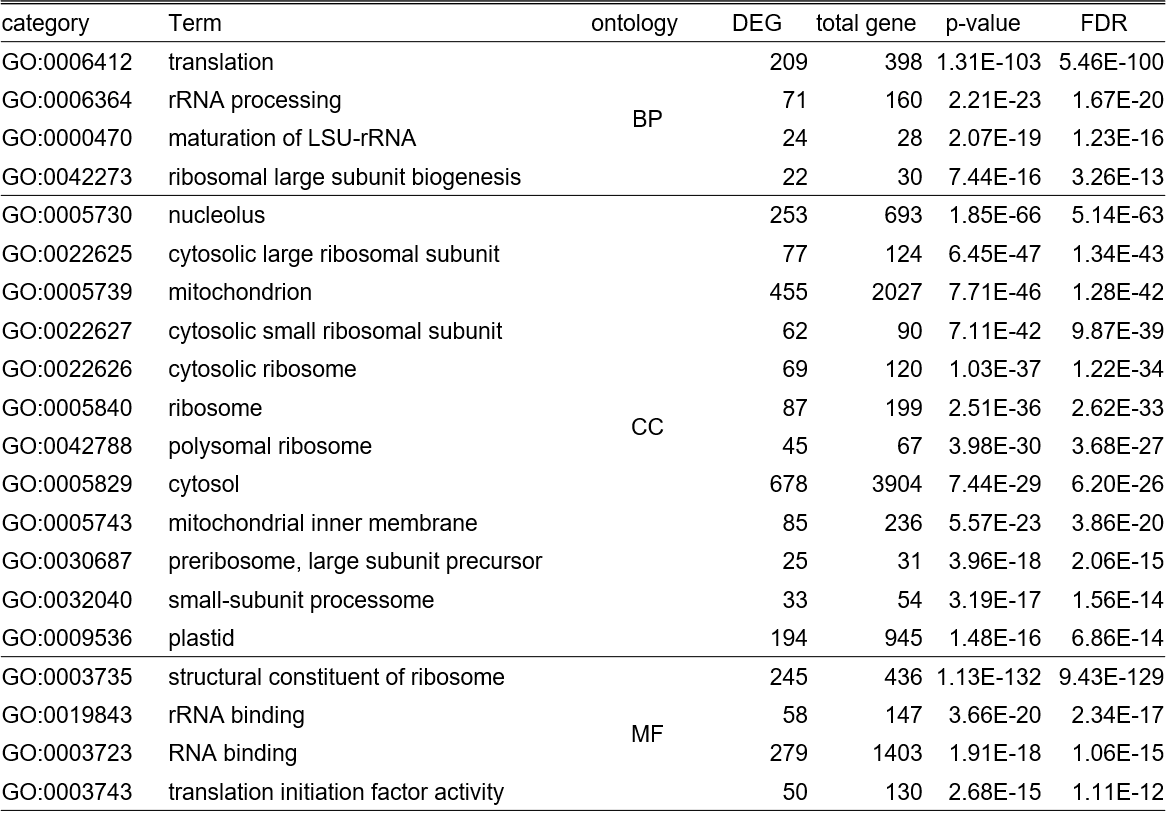
**

Twenty GO terms with the lowest False Discovery Rate (FDR) were listed.

**Supplementary Table 2. Top 20 GO terms detected by GO enrichment analysis of up-regulated genes in 2 d compared with in 0 d in ‘ON’.**


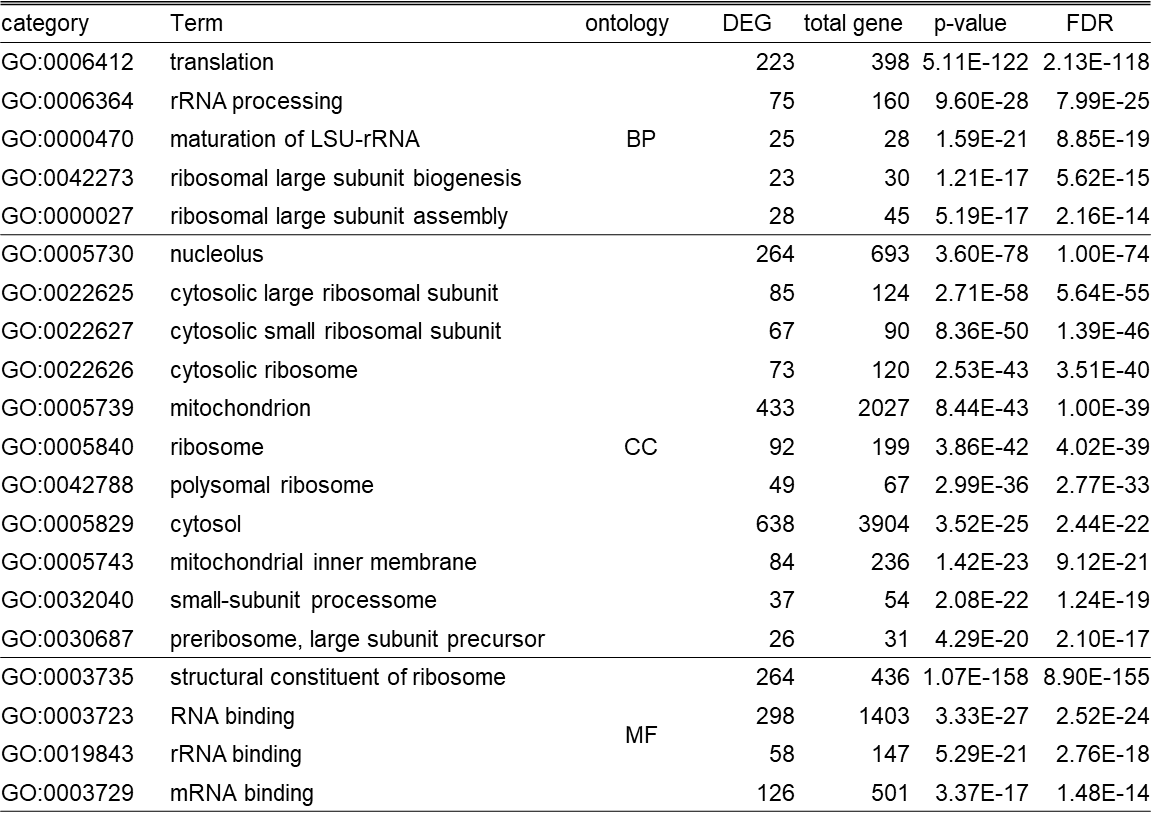


Twenty GO terms with the lowest False Discovery Rate (FDR) were listed.

**Supplementary Table 3. GO enrichment analysis of cluster A.**


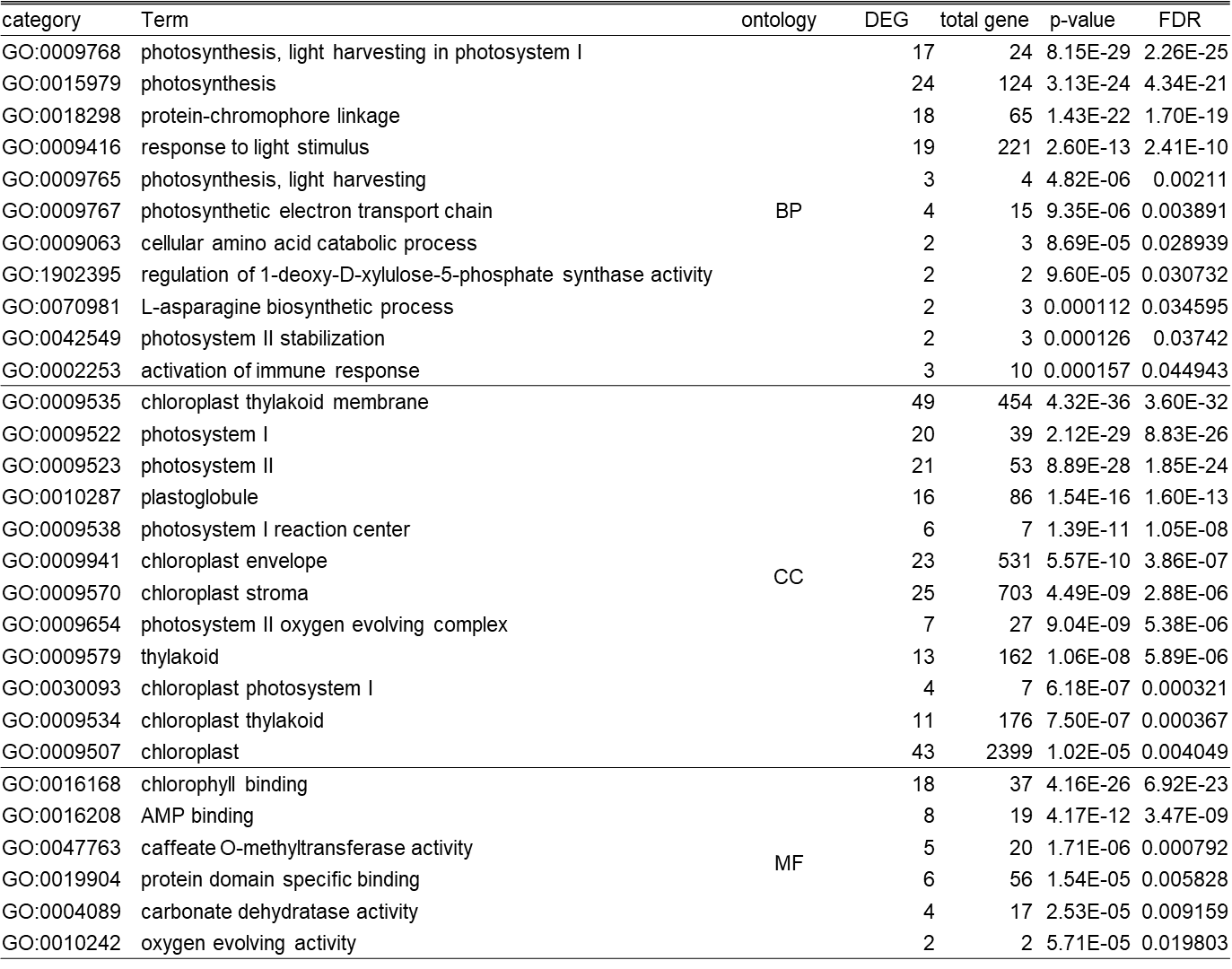


GO terms with FDR<0.05 were listed.

**Supplementary Table 4. GO enrichment analysis of cluster C.**


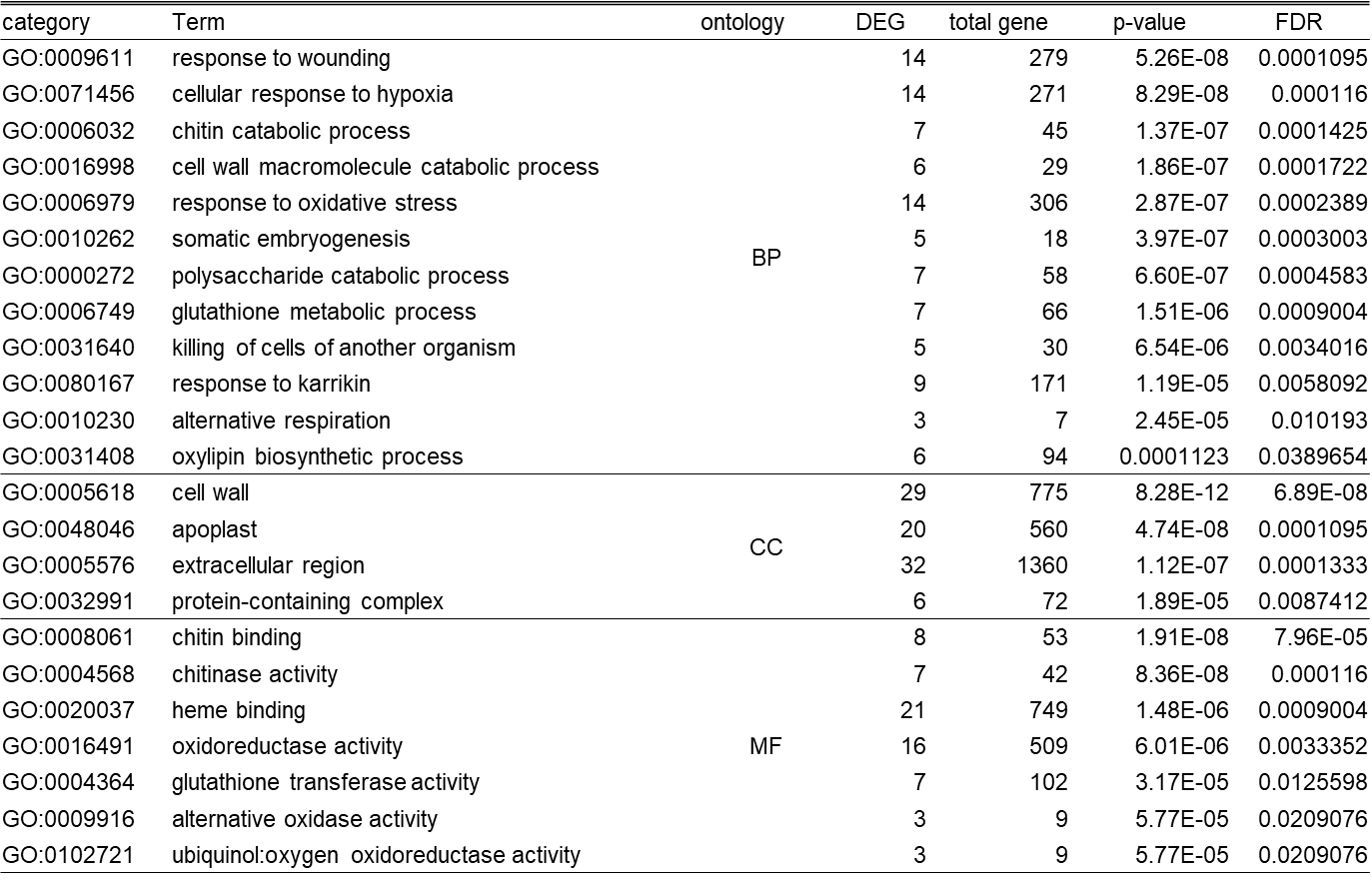


GO terms with FDR<0.05 were listed.

**Supplementary Table 5. GO enrichment analysis of cluster D.**


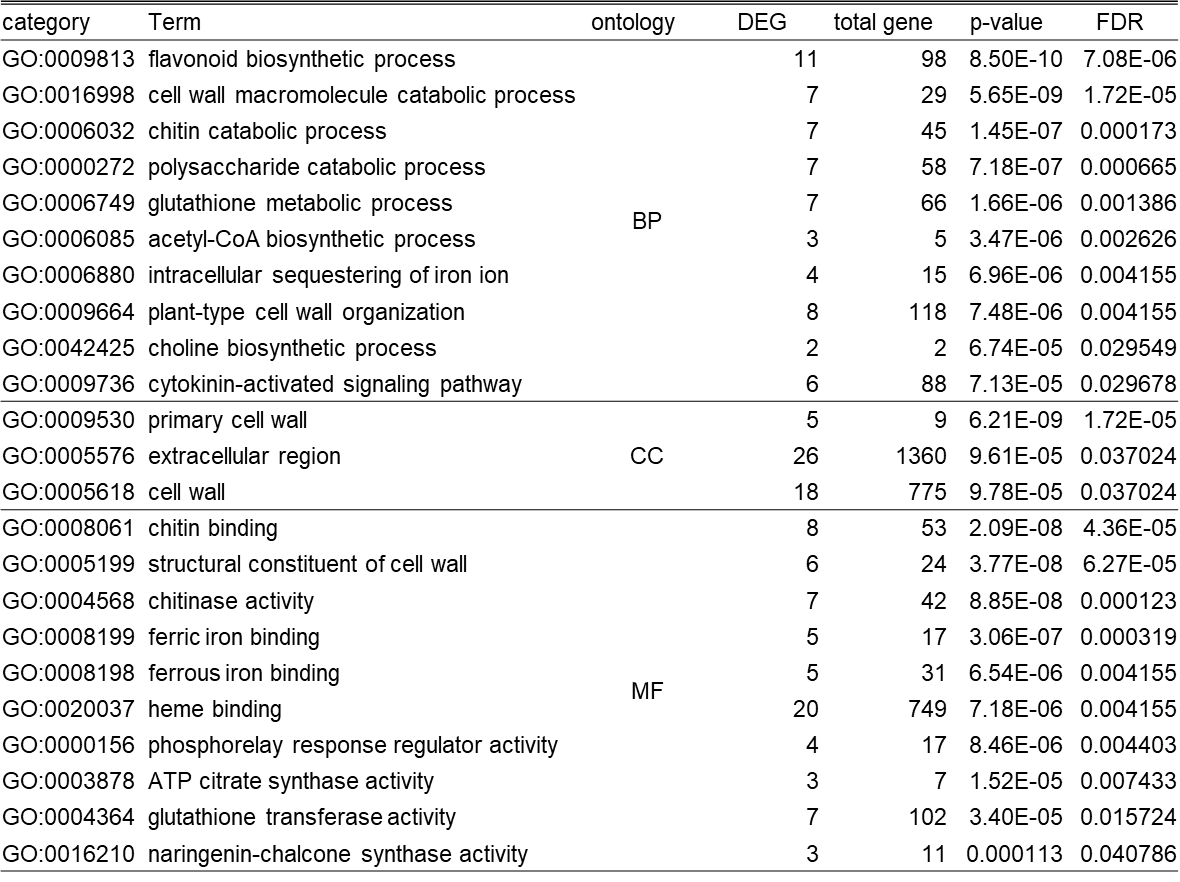


GO terms with FDR<0.05 were listed.

**Supplementary Table 6. GO enrichment analysis of cluster F.**


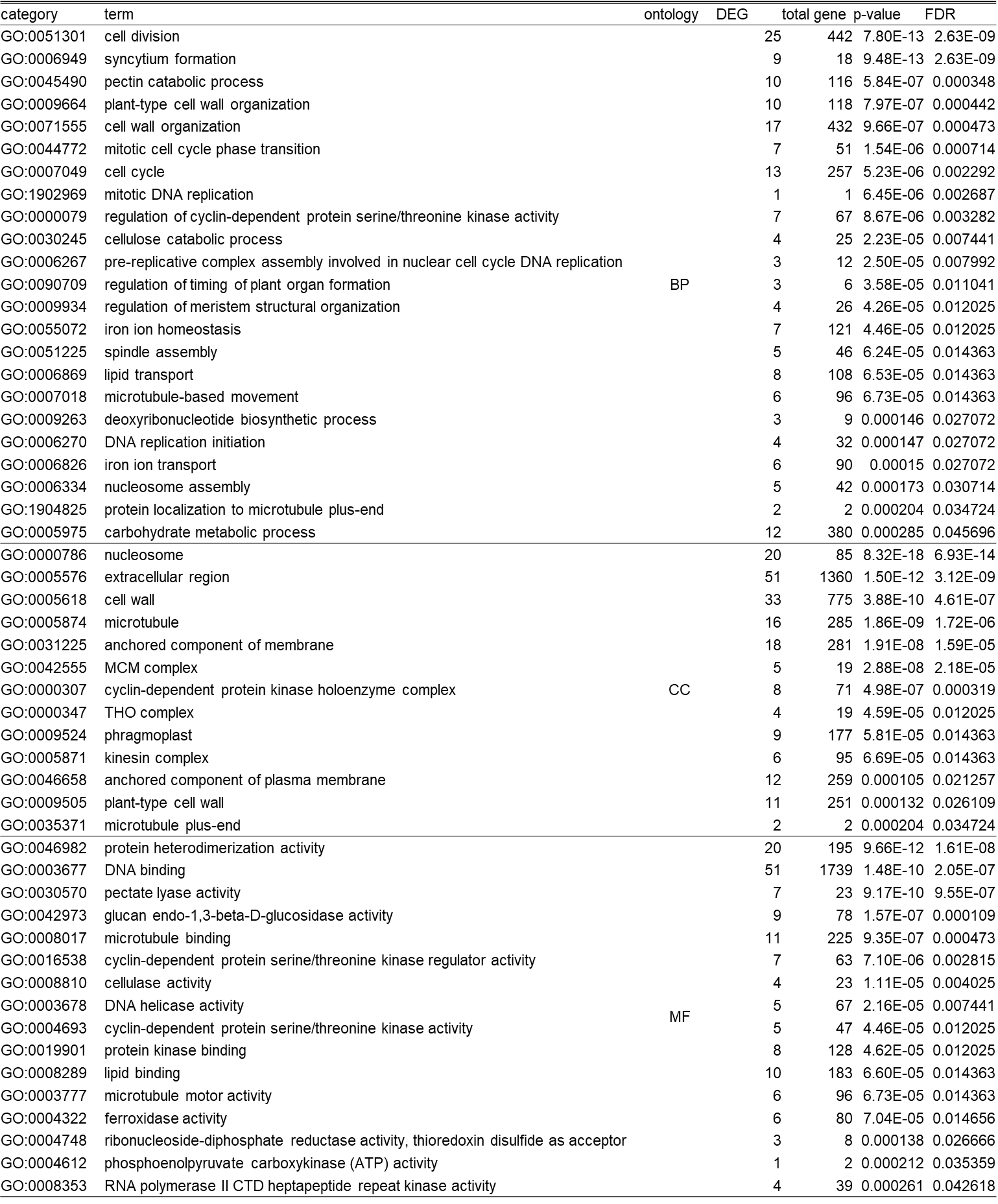


**Supplementary Table 7. Sequence information for the primers used in this study.**

| **Name** | **Sequence** | **Applications** |
| --- | --- | --- |
| pPLV26-VcESR_F | 5'-CCTCGAGCTAGTTGGAATAGGTTATGGAAGAAGCCCTGAGAAG-3' | For constructing pPLV26-35S:VcESR |
| pPLV26-VcESR_R | 5'-GCTGCAGTATGGAGTTGGGTTTTAAGCATTCTGCATATTTTCAG-3' | For constructing pPLV26-35S:VcESR |
| pPLV26_nptII_F | 5'-GAAGTGCCGGGGCAGGATCTCC-3' | For amplifying nptII gene |
| pPLV26_nptII_R | 5'-GATATTCGGCAAGCAGGCATCG-3' | For amplifying nptII gene |
| VcESR_expression_F | 5'-TCACGGTAACTTCCCTGTGC-3' | For qPCR analysis to detect *VcESR* expression |
| VcESR_expression_R | 5'-GACCTCCTCCAAAAGACCCG-3' | For qPCR analysis to detect *VcESR* expression |
| VcWUS_expression_F | 5'-GTGGACACCAACAACTGAGC-3' | For qPCR analysis to detect *VcWUS* expression |
| VcWUS_expression_R | 5'-CTTCTTCTGCCTCTCACGAG-3' | For qPCR analysis to detect *VcWUS* expression |
| VcUBC28_F | 5’-CCATCCACTTCCCTCCAGATTATCCAT-3’ | For qPCR analysis to detect reference gene expression |
| VcUBC28_R | 5’-ACAGATTGAGAGCAGCACCTTGGA-3’ | For qPCR analysis to detect reference gene expression |

**
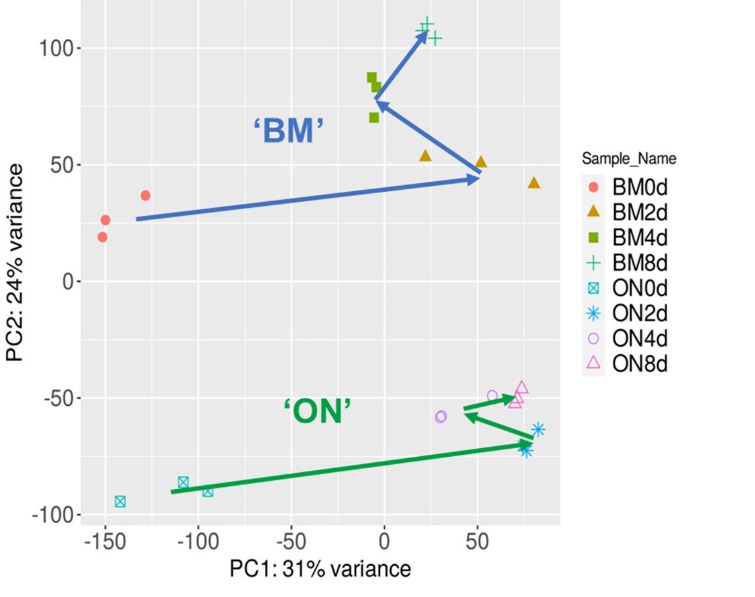
**

**Supplementary Figure 1. Principal component analysis (PCA) of RNA-seq data.**

The TPM values of each sample were subjected to PCA using the R prcomp function. Periodical changes in ‘BM’ and ‘ON’ were indicated by blue and green arrows, respectively.


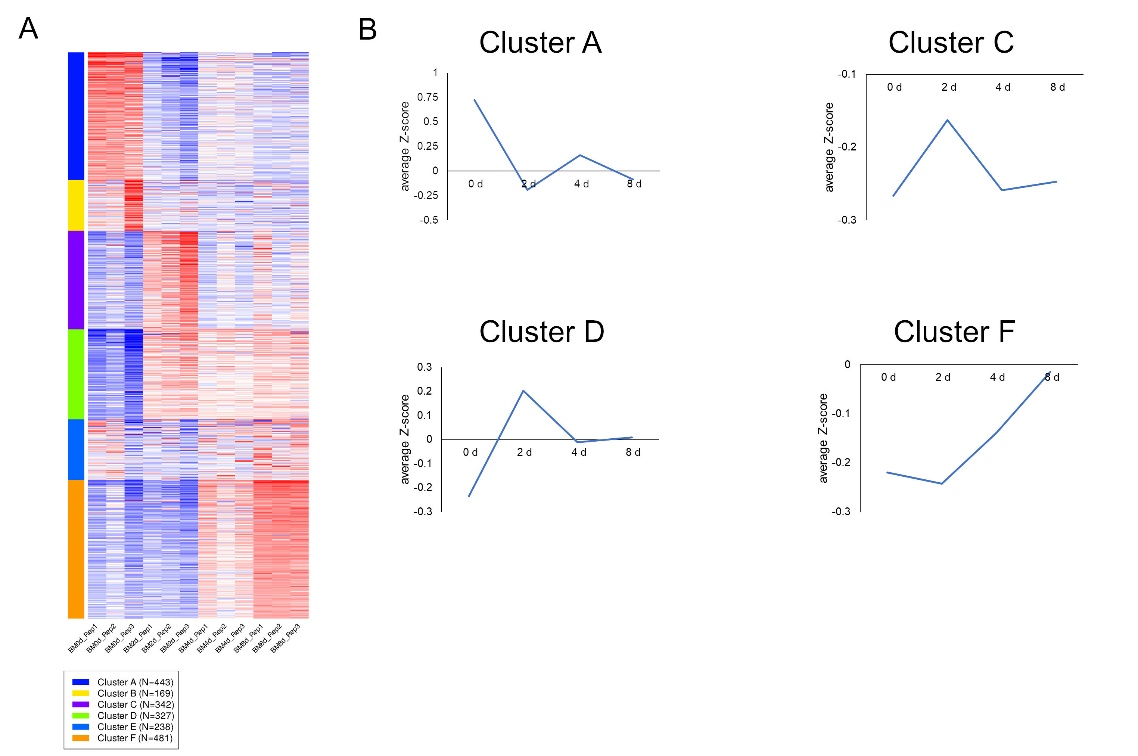


**Supplementary Figure 2. K-means clustering of genes expressed in ‘BM’.**

(A) Clustering of genes expressed in ‘BM’ based on expression pattern.

Genes were ranked by standard deviation between all samples and the top 2000 genes were used for k-means clustering. Red means high Z-score while blue means low Z-score. The figure was drawn by using iDEP website (Ge et al, 2018). (B) Expression pattern of genes assigned to each cluster. Z-scores were calculated from the data matrix of mean TPM.


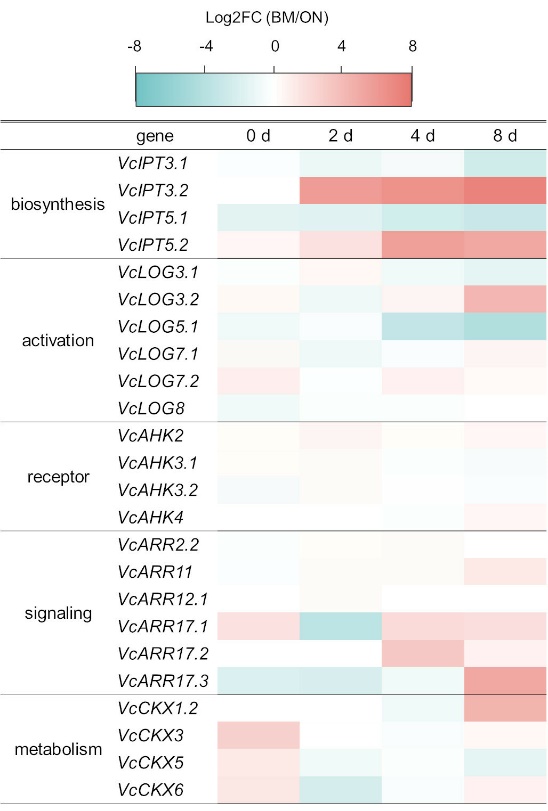


**Supplementary Figure 3. Heatmap illustrating log2FC(BM/ON) of cytokinin-related genes.**

Red indicates higher expression in ‘BM’ compared to ‘ON’, while blue indicates lower expression in ‘BM’ compared to ‘ON’. Genes include *VcISOPENTENYLTRANSFERASE* (*VcIPT*), *VcLONELY GUY* (*VcLOG*), *VcARABIDOPSIS HISTIDINE KINASE* (*VcAHK*), *VcARABIDOPSIS RESPONSE REGULATORS* (*VcARR*), and *VcCYTOKININ OXIDASE*/*DEHYDROGENASE* (*VcCKX*). Genes with TPM > 1 were selected.


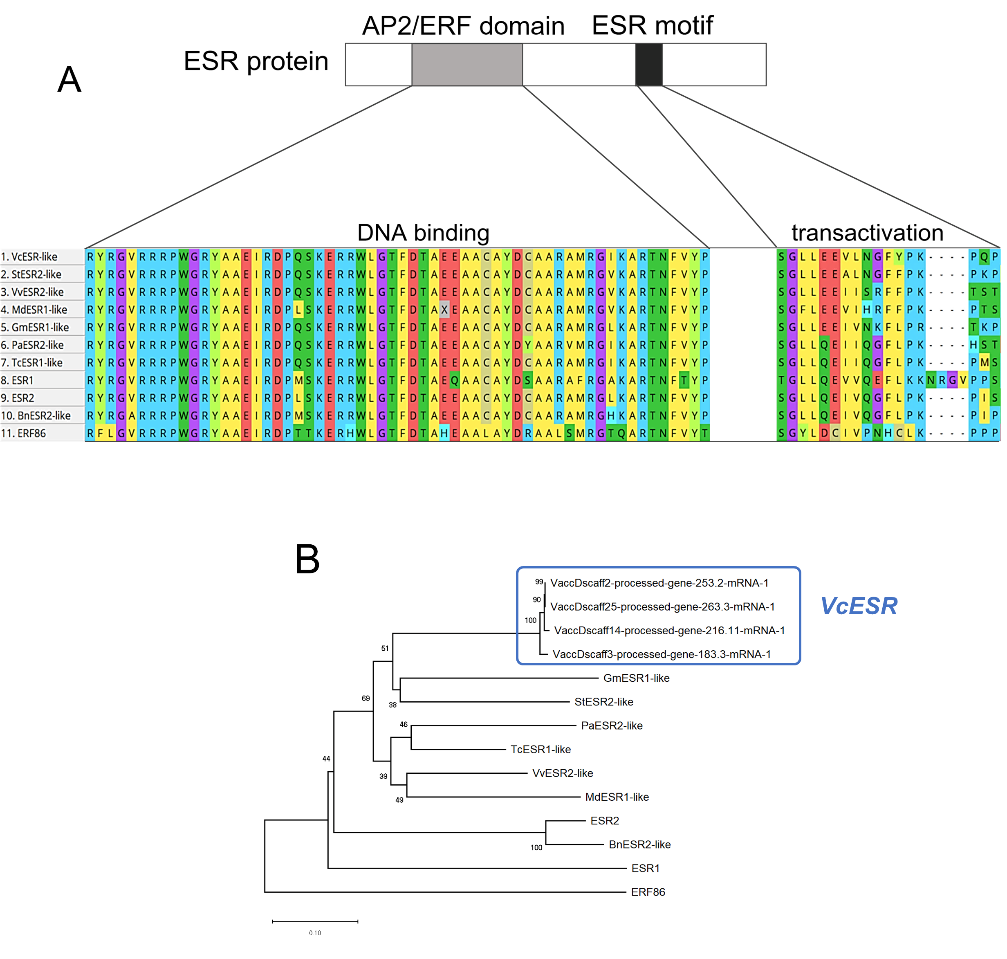


**Supplementary Figure 4. Comparison of partial amino acid sequences and phylogenetic analysis of ESR-like proteins from different plant species**

(A) Structural characteristics and alignment of amino acid sequences of ESR-like proteins from different plant species. ESR family proteins contain DNA binding AP2/ERF domain and transactivating ESR motif. The GenBank accession numbers are as follows. 1.VcESR-like: *Vaccinium corymbosum* ESR-like (VaccDscaff2-processed-gene-253.2-mRNA-1) 2.StESR2: *Solanum tuberosum* ESR2-like (XP_006357626) 3.VvESR2-like: *Vitis vinifera* ESR2-like (XP_002271778) 4.MdESR1-like: *Malus domestica* ESR1-like (XP_008390095) 5.GmESR1-like: *Glycine max* ESR1-like (AFO52509) 6.PaESR2-like: *Populus alba* ESR2-like (XP_034909885) 7.TcESR1-like: *Theobroma cacao* ESR1-like (XP_007044810) 8.ESR1: *Arabidopsis thaliana* ESR1 (AAL56226) 9.ESR2: *Arabidopsis thaliana* ESR2 (NP_173864) 10.BnESR2-like: *Brassica napus* ESR2-like (XP_013652850) 11.ERF86: *Arabidopsis thaliana* Ethylene-responsive transcription factor (ERF086) (NP_197357) .

(B) Phylogenetic tree of ESR family proteins constructed by neighbor-joining method with the program MEGA X. The scale indicates the average substitutions per site. Tetraploid highbush blueberry genome has four alleles of *VcESR* with 98-99% sequence similarity.
